# Validation of a surgical drill with a haptic interface in spine surgery

**DOI:** 10.1038/s41598-023-27467-w

**Published:** 2023-01-12

**Authors:** Kento Yamanouchi, Shunya Takano, Yuichiro Mima, Takuya Matsunaga, Kouhei Ohnishi, Morio Matsumoto, Masaya Nakamura, Tomoyuki Shimono, Mitsuru Yagi

**Affiliations:** 1 Department of Orthopaedic Surgery, Keio University School of Medicine, 35 Shinanomachi, Shinjyuku, Tokyo, Japan; 2 Kanagawa Institute of Industrial Science and Technology, Kawasaki, Japan; 3 Keio Frontier Research and Education Collaborative Square, Keio University, Tokyo, Japan; 4 Faculty of Engineering, Yokohama National University, 79-5 Tokiwadai, Hodogaya-Ku, Yokohama, 240-8501 Japan

**Keywords:** Medical research, Biomedical engineering

## Abstract

Real haptics is a technology that reproduces the sense of force and touch by transmitting contact information with real objects by converting human movements and the feel of the objects into data. In recent years, real haptics technology has been installed in several surgical devices. A custom-made surgical drill was used to drill into the posterior lamina to verify the time required for penetration detection and the distance the drill advanced after penetration. A surgeon operated with the drill and the same aspects were measured and verified. All experiments were performed on female miniature pigs at 9 months of age with a mean body weight of 23.6 kg (range 9–10 months and 22.5–25.8 kg, n = 12). There were statistically significant differences in the average reaction time and the distance travelled after penetration between a handheld drill and the drill with the penetration detection function (p < 0.001). The reaction time to detect penetration and the distance after penetration were both significantly improved when compared with those of the handheld surgical drill without the penetration detection function, with mean differences of 0.049 ± 0.019 s [95% CI 0.012, 0.086 s] and 2.511 ± 0.537 mm [95% CI 1.505, 3.516 mm]. In this study, we successfully conducted a performance evaluation test of a custom-made haptic interface surgical drill. A prototype high-speed drill with a haptic interface accurately detected the penetration of the porcine posterior lamina.

## Introduction

As a treatment for various musculoskeletal diseases, spine surgery involves drilling into the bone near the spinal nerves and vascular organs. This is an extremely demanding procedure because it involves the manipulation of hard tissue in the vicinity of highly vulnerable soft tissue. As a result, intraoperative complications of spine surgery occur at a certain rate. Bone drilling is usually performed with a high-speed drill, and severe complications, such as dural lacerations and spinal cord injury, may occur during the operation. Neurological complications are devastating and can severely diminish a patient's quality of life due to loss of motor function, limiting the patient’s functional ability and causing excruciating neurological pain; every effort should be made to prevent the occurrence of such complications.

Previous reports have shown that the complication rate of spine surgery is relatively high, ranging from 1 to 17%^1–6^. Among these surgical complications, dural lacerations have been reported to affect 2–13% of cases. Most dural lacerations are caused by bone drilling during decompression of the spinal cord^7^. It has also been reported that the incidence of intraoperative complications, such as nerve injury, is inversely related to the number of years of surgical experience of the surgeon. Imajo et al. found that spine surgeons with less than 5 years of surgical experience had the highest frequency of complications^1^. It is widely accepted that in spine surgery, when drilling bone using a high-speed drill, the surgeon relies on the feel of the drill to determine whether the bone has been penetrated. Therefore, it is thought that the number of years of surgical experience affects the surgeon’s ability to detect bone penetration, and this directly affects the incidence of complications.

In contrast, Imajo et al. described the results of a survey of spine surgery complications and concluded that the incidence of intraoperative dural injuries was similar between spine surgeons with less than 10 years of experience and those with more than 10 years of experience^1^. In other words, although the number of years of experience in spine surgery influences the complication rate to some extent due to the nature of the procedure, dural lacerations and neurological injuries still occur regardless of the number of years of experience.

In recent years, real haptics technology, which has been utilized in various fields, has been installed in high-speed drills to establish a surgical method with greater safety^8,9^. Real haptics is a technology that reproduces the sense of force and touch by transmitting contact information with real objects and the surrounding environment in both directions by converting human movements and the feel of the objects into data. By converting movements and textures into data, it is possible to program force tactile sensation data into machines, adjust the degree of force, and transmit force tactile sensation over a long distance^9^. Specifically, by monitoring the drilling force in real time when using the drill, it will be possible to detect penetration, automatically stop the drill, and proceed with the surgery while obtaining detailed information, such as the bone hardness and the identity of the drilling layer. In addition, by extracting intraoperative drilling sensation and movement data, we believe that such data can be applied to develop a training simulator that reproduces a realistic sensation of force and touch and adds an assistance function based on the movements of a skilled surgeon.

**Figure 1:**
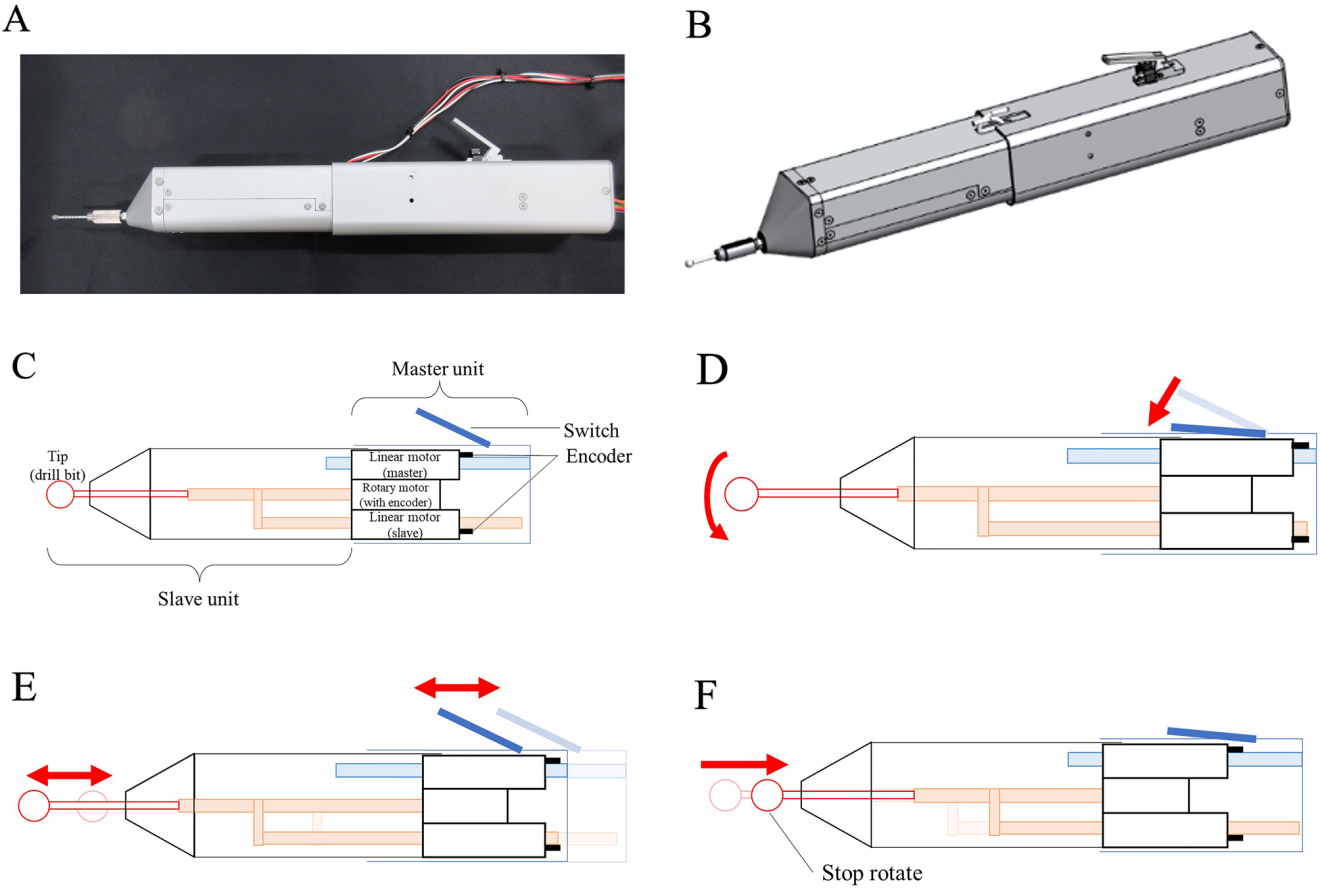
Custom-made surgical drill. (**A**) Clinical image of the custom-made surgical drill. (**B**) Schematic view of the custom-made surgical drill. (**C**) Description of the control unit of the custom-made surgical drill. (**D**) Schematic view of the switching motion. (**E**) Schematic view of the bilateral motion. (**F**) Schematic view of the automatic stop function after detecting penetration.

We hypothesized that a prototype high-speed drill with a haptic interface could detect the penetration of the porcine posterior lamina more accurately and more reproducibly than experienced spine surgeons could recognize (Fig. 1A,B). In this study, the safety, efficacy, and reproducibility of the previously described prototype high-speed drill with a haptic interface were evaluated using a porcine spine, which is histologically similar to the human bone structure and similar in strength. Confirmation of force-tactile transmission under bilateral control and penetration detection experiments were conducted.

## Materials and methods

### Custom-made surgical drill with a haptic interface

The details of the haptic drill are shown elsewhere^10^. The custom-made drill has a master–slave integrated structure and is driven by a linear motor with two optical encoders and a rotary motor with a rotary encoder^10^. A schematic drawing of the prototype is shown in Fig. 1C. The rotary motor is mounted on the shaft part as the mover of the linear motor on the slave side. The other linear motor, as the master side, is connected to the switch part. The maximum rotation speed of the rotary motor is 60,800 RPM. Proportional velocity control based on robust acceleration control by a disturbance observer is applied to the rotary motor. The rotary motor is rotated while the surgeon pushes down the switch (Fig. 1D). Linear encoders measure the position change of the mover part of the linear motor. The reaction force is estimated by a disturbance observer. The two linear motors are controlled by real haptics to synchronously move in parallel to the rotary axis of the rotary motor used to cut the environment with the drill bits (Fig. 1E). In addition, the action force applied by the surgeon to the master part is matched to the reaction force from the cutting object applied to the slave part. As a result, the haptic sensation can be transmitted between the master part and slave part^10^. Moreover, when the custom-made surgical drill detects penetration, position control is applied to the linear motor on the slave side to pull in the drill bit while the rotary motor is stopped automatically (Fig. 1F).

### Control method

**Figure 2:**
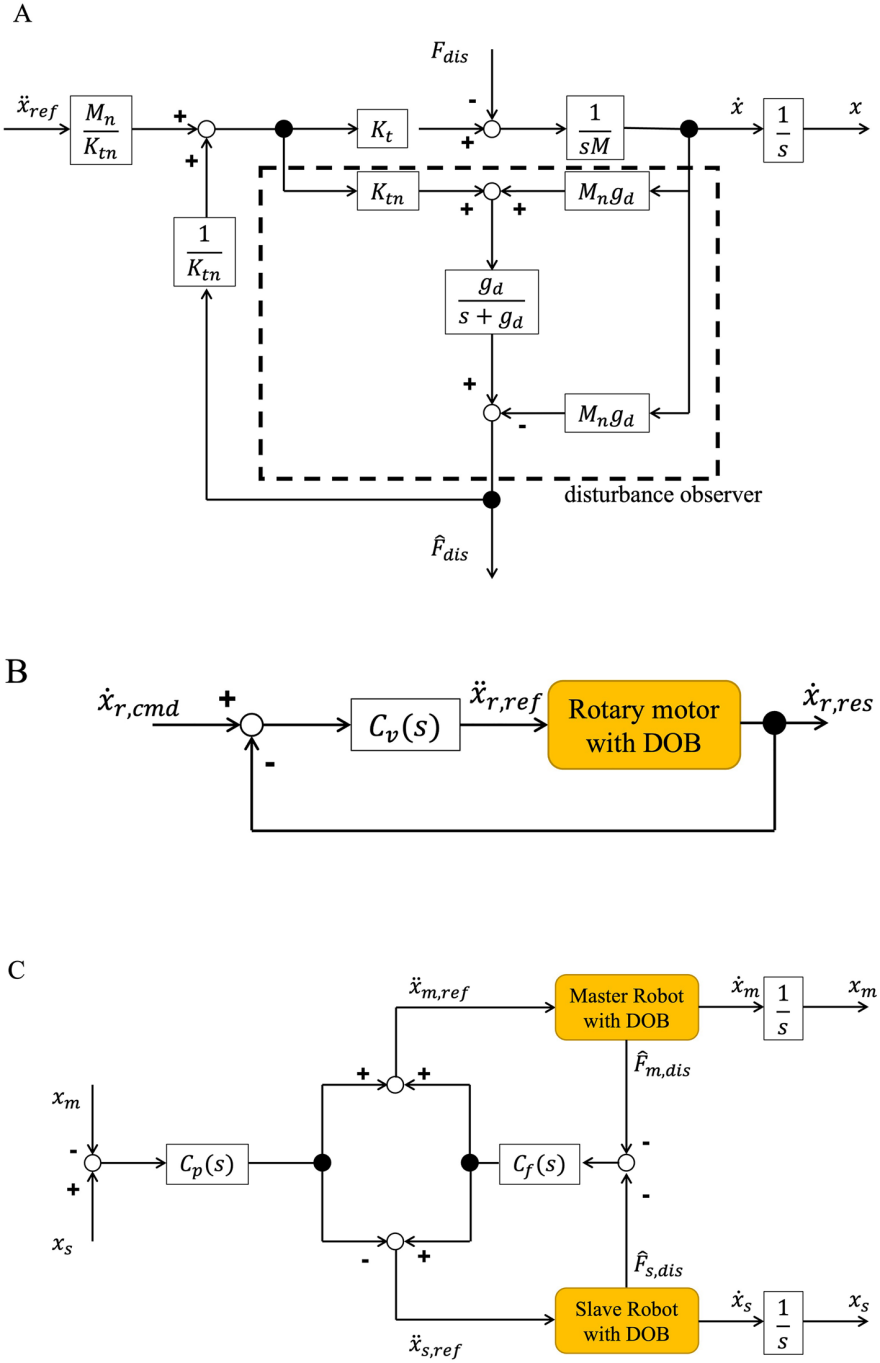
Block diagram. (**A**) Block diagram of the disturbance observer. (**B**) Block diagram of the velocity control. (**C**) Block diagram of the bilateral control.

The custom-made surgical drill is controlled based on robust acceleration control with a disturbance observer (DOB). The block diagram of the DOB is shown in Fig. 2A. ${\ddot{x}}_{ref}$, *K*_*t*_*, M, *_*n*_*, *${F}_{dis}$*, *${\widehat{F}}_{dis}$*,* and ${g}_{d}$ are the acceleration reference, force constant, motor mass, nominal value, disturbance force from the environment, estimated disturbance force, and cut-off frequency of the low-pass filter, respectively. The disturbance from the environment is estimated by comparison between the command value and the response value. This compensation makes the bilateral, position, and velocity control robust. Additionally, a low-pass filter is applied to remove noise.

The rotary motor is controlled by velocity control. Figure 2B shows the block diagram of the velocity control. ${\dot{x}}_{r,cmd}$, ${C}_{v}(s)$, and ${\dot{x}}_{r,res}$ are the velocity command of the rotary motor, velocity controller, and velocity response of the rotary motor, respectively. The rotary motor is rotated while the surgeon pushes down the switch. In contrast, the rotary motor is stopped when the switch is not pushed down or the drill detects penetration. Thus, when the rotation speed is set at 30,000 rpm, the velocity command ${\dot{x}}_{r,cmd}$ $\mathrm{rad}/\mathrm{s}$ is defined as (1).

1 $${\dot{x}}_{r,cmd}=\left\{\begin{array}{l}1000\pi \left(\mathrm{switch \; on}\right) \\ 0 \left(\mathrm{switch \; off \; or \; detect \; penetration}\right).\end{array}\right.$$

Bilateral control is applied to the two linear motors to realize transmission of the haptic sensation. The block diagram of the bilateral control is shown in Fig. 2C. *m, s, C*_*p*_(*s*)*,* and *C*_*f*_(*s*) are the master part, slave part, position controller, and force controller, respectively. ${x}_{m}$ and ${x}_{s}$ are the measurements of the linear encoder. In bilateral control, two equations are realized:

2 $${x}_{m}-{x}_{s}=0,$$

3 $${F}_{m}+{F}_{s}=0.$$

These equations represent the law of action and reaction. Thus, the haptic sensation between the two linear motors is realized by bilateral control.

### Methods to detect penetration

To detect penetration, the differential value of the position and reaction force are used^11^. In the drilling state, the reaction force from the cutting object is applied to the slave part. In contrast, when the drill penetrates the object, the reaction force is decreased. Additionally, the velocity of the slave part is increased due to the decrease in the reaction force. Therefore, large displacements of position and reaction force are generated by penetration. Thus, the drill detects the penetration when Eqs. (4) and (5) are satisfied.

4 $${\dot{x}}_{s,pene}>{\dot{x}}_{threshold},$$

5 $${\dot{F}}_{s,pene}<{\dot{F}}_{threshold}.$$

${\dot{x}}_{s,pene}$ and ${\dot{F}}_{s,pene}$ are the velocity and the differential value of the reaction force of the slave part, respectively. ${\dot{x}}_{threshold}$ and ${\dot{F}}_{threshold}$ are threshold values. ${\dot{x}}_{s,pene}$ and ${\dot{F}}_{s,pene}$ are estimated by Eqs. (6) and (7).

6 $${\dot{x}}_{s,pene}=\frac{s\cdot {g}_{pene}}{s+{g}_{pene}} {x}_{s},$$

7 $${\dot{F}}_{s,pene}=\frac{{s\cdot g}_{pene}}{s+{g}_{pene}} {\widehat{F}}_{dis}.$$

${g}_{pene}$ is the cut-off frequency of the low-pass filter.

When the custom-made surgical drill detects penetration, position control is applied to the linear motor on the slave side to pull in the drill bit. Additionally, the rotary motor is stopped automatically.

### Animals and surgical procedures

After review and approval by the Judging Committee of Experimental Animal Ethics of Keio University School of Medicine, all experiments were performed on female miniature pigs at approximately 9 months old with a mean body weight of 23.6 kg (range 9–10 months and 22.5–25.8 kg, n = 12). All animals were purchased from the laboratory (Kagoshima Miniature Swine Research Center, Kagoshima, Japan) and housed and treated in accordance with rules approved by the Ethics Committee (no. 18047). All experiments were performed in accordance with any relevant guidelines and regulations. This study follows the recommendations in the ARRIVE guidelines.

The miniature pigs undergoing surgery were premedicated with midazolam (Dormicum, 0.1 mL/kg, Hoffmann-La Roche AG, Switzerland) administered subdermally. Subsequently, inhalant anaesthetics and isoflurane (Isiflu, Dainipon-sumitomo, Osaka, Japan) were used with maintenance at a 2% flow rate after orotracheal intubation. All pigs were killed immediately after surgery by an overdose of isoflurane. After establishing anaesthesia, a standard midline dissection was performed by 1 of 3 board-certified spine surgeons using electrocautery, and the laminae of the thoracic vertebrae and lumbar vertebrae were carefully exposed.

### Placement of the surgical drill with a haptic interface

**Figure 3:**
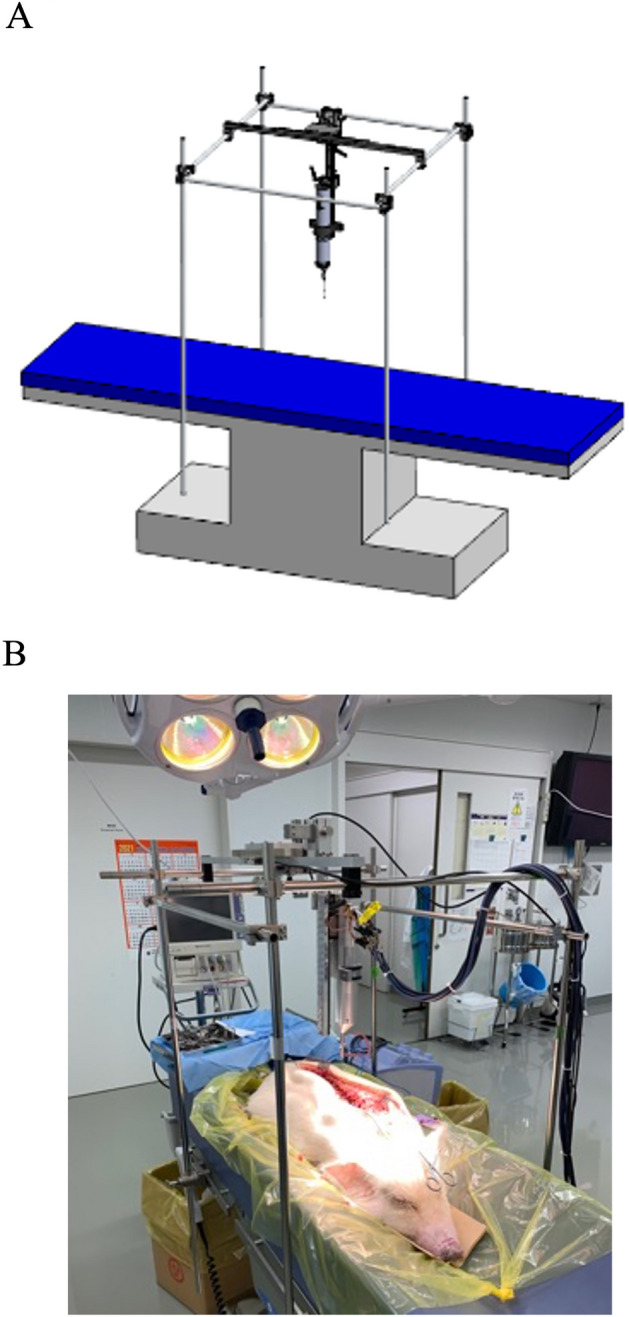
Schematic view of a custom-made surgical table for the animal experiments. (**A**) The custom-made surgical drill was placed directly above the surgical table via a post. (**B**) Intraoperative view of the animal experiment.

The experimental setup is shown in Fig. 3A,B. The custom-made surgical drill was placed directly above the surgical table via a post (Fig. 3A,B). Then, the whole back of the miniature pig was dissected, and the thoracic and lumbar vertebrae were exposed on the table. The custom-made surgical drill was lowered perpendicularly to the laminae of the miniature pig until the tip of the burr proceeded to the lamina. The touch of the tip to the lamina was confirmed by direct visualization. Next, force-tactile sensation transmission under bilateral control was confirmed, and the experiments were conducted. The velocity command of the rotary motor ${\dot{x}}_{r,cmd}$ was set to 30,000 RPM. The slave speed (drill cutting speed) was set to 0.25 mm/s. A steel burr with a diameter of 5.0 mm was used in all experiments (reference #5820-010-240, TPS Elite round fluted aggressive, Stryker Instruments MI, USA). Each burr was used only once and then discarded.

### Penetration of the laminae using a surgical drill with a haptic interface

First, the custom-made surgical drill was used to drill the posterior lamina without the penetration detection function. After penetration was detected, the threshold was set as a control, where − 7 N/s was ${\dot{F}}_{threshold}$ and 2 mm/s was ${\dot{x}}_{threshold}$.

The time required for penetration detection was defined as the time from the start of the decrease in the reaction force of the slave until ${\dot{F}}_{s,pene}$ exceeded − 7 N/s and ${\dot{x}}_{s,pene}$ exceeded 2 mm/s.

After that, the penetration detection function was turned on, and the measurement results of the drilling of the posterior laminae were used to verify:

1. The time required for penetration detection.
2. The distance advanced by the drill after penetration.

**Figure 4:**
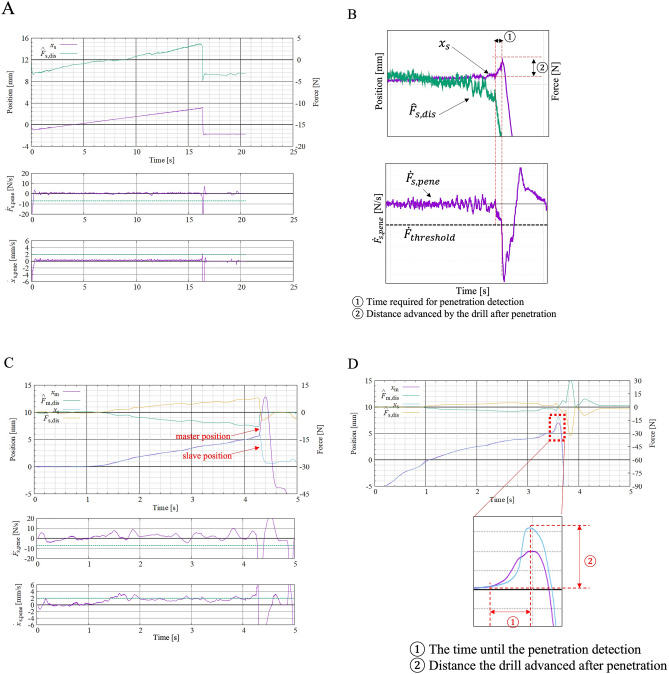
Position and force–time waveform of the master and slave units on the haptic drill. (**A**) The distance the drill advanced after penetration was defined as the distance that the slave advanced before the device detected the penetration and started retracting the drill. (**B**) This is a magnified view of the waveform, especially during penetration. The horizontal axis shows time, and the vertical axis shows reaction force. The waveforms are magnified to emphasize the changes in the waveforms during penetration. ① Shows the time required for penetration detection. ② Shows the distance advanced by the drill after penetration. (**C**,**D**) The distance travelled after penetration was defined as the distance travelled from "the position where the drill started to move forwards rapidly" to "the peak of the drill (slave) position", i.e., the distance travelled until the surgeon detected the penetration and started to retract the drill body. ① shows the time required for penetration detection. ② shows the distance advanced by the drill after penetration.

The distance the drill advanced after penetration was defined as the distance that the slave advanced before the device detected the penetration and started retracting the drill (Fig. 4A,B).

Next, the surgeon operated the drill without the penetration detection function and verified:

1. The time required for penetration detection.
2. The distance advanced by the drill after penetration.

The time until the surgeon recognized the penetration detection was set as the reaction time, which was defined as "the time when the drill position started to move forwards rapidly" to "the time when the drill stopped moving forwards (the time when the speed was displaced negatively)", that is, the time taken by the surgeon to detect the penetration and start retracting the drill body. The participants were instructed to move the master in the opposite direction of penetration (retracting the drill).

The distance travelled after penetration was defined as the distance travelled from "the position where the drill started to move forwards rapidly" to "the peak of the drill (slave) position", i.e., the distance travelled until the surgeon detected the penetration and started to retract the drill body (Fig. 4C,D).

Finally, the haptic drill with the penetration detection function was used by the surgeon, and the same items were measured and verified.

### Penetration of laminae using a surgical drill with a haptic interface using horizontal positional information

Next, to assess if the haptic drill is able to detect the penetration of lamina in an actual surgery setting, the surgeon operated the haptic drill with horizontal movement similar to the actual surgical procedure (see Supplementary Video S1).

The surgeon operated:

1. The drill using the horizontal position information.
2. The drill fixed diagonally to the spine.

**Figure 5:**
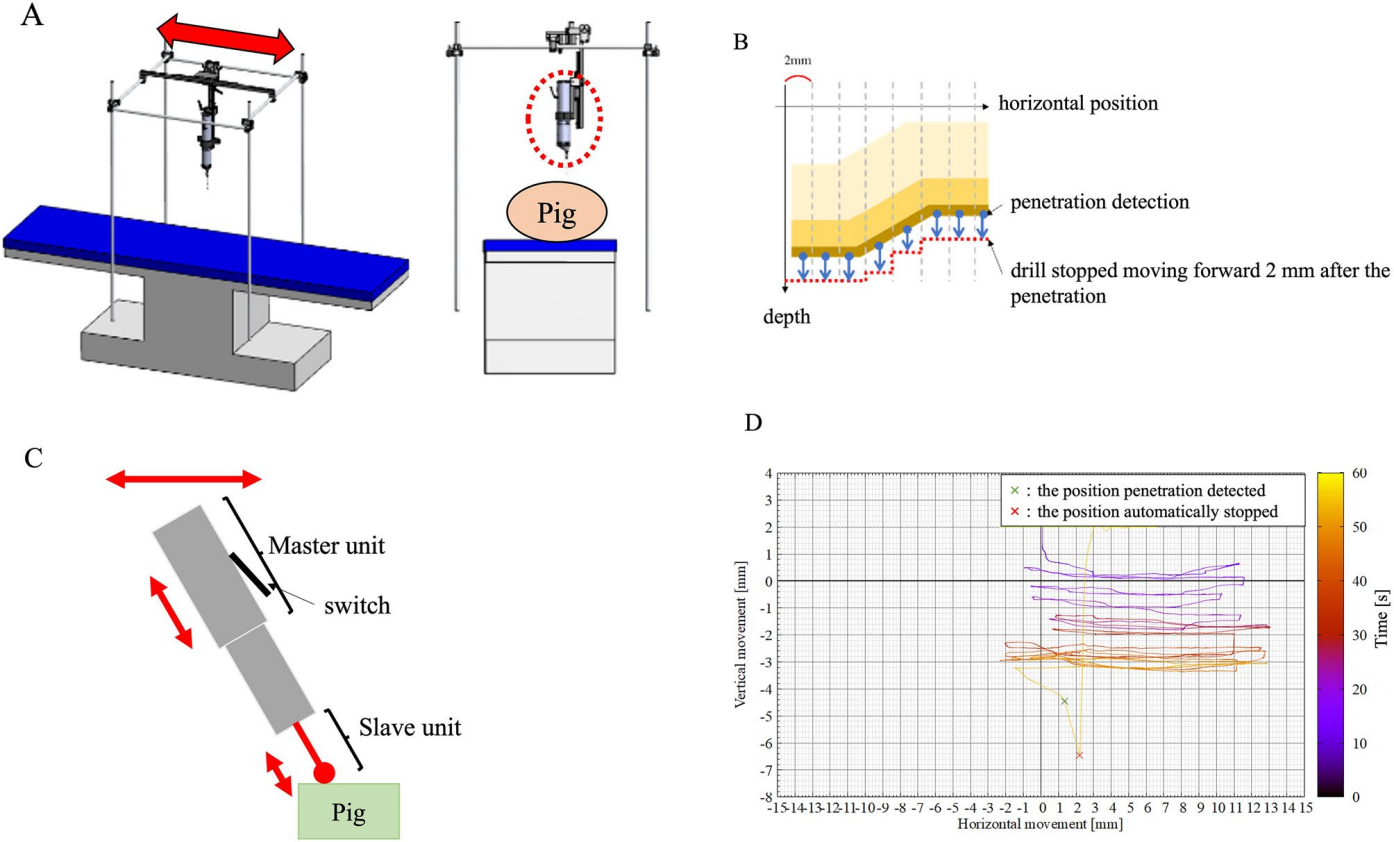
Schematic view of the surgical drill with a haptic interface using horizontal positional information. (**A**) Custom-made surgical table for the haptic drill in the horizontal direction. (**B**) The horizontal position was divided into 2 mm increments, and penetration detection was performed within each section. The drill stopped moving forwards 2 mm after penetration. (**C**) The surgeon horizontally operated the haptic drill installed diagonally to the spine. (**D**) Position waveform of the haptic drill using horizontal position information. The waveform colour changes from black to yellow over time.

To verify accurate penetration detection according to the shape of the spine, the horizontal position was divided into 2 mm increments, and penetration detection was performed within each section (Fig. 5A,B). In addition, the surgeon horizontally operated the haptic drill installed diagonally to the spine (Fig. 5C,D).

During the experiments, the haptic drill was placed firmly on a post on the surgical table to eliminate any effects of vibration. All experiments were performed by 3 board-certified senior spine surgeons. All drilling was performed three times by each of the three participants.

### Statistical analysis

We calculated the overall summary statistics, including the means and standard deviations for continuous variables and the frequencies and percentages for categorical variables. The mean difference between the abovementioned groups was calculated with a 95% confidence interval (CI). A p value less than 0.05 with a CI of 95% was considered statistically significant. A p value less than 0.05 was considered statistically significant. Data were analysed with the Statistical Package for the Social Sciences (SPSS statistics version 27.0, IBM Corp., Armonk, NY).

## Results

### The time to detect lamina penetration and the travel distance after penetration of the haptic interface surgical drill with the penetration detection function

**Figure 6:**
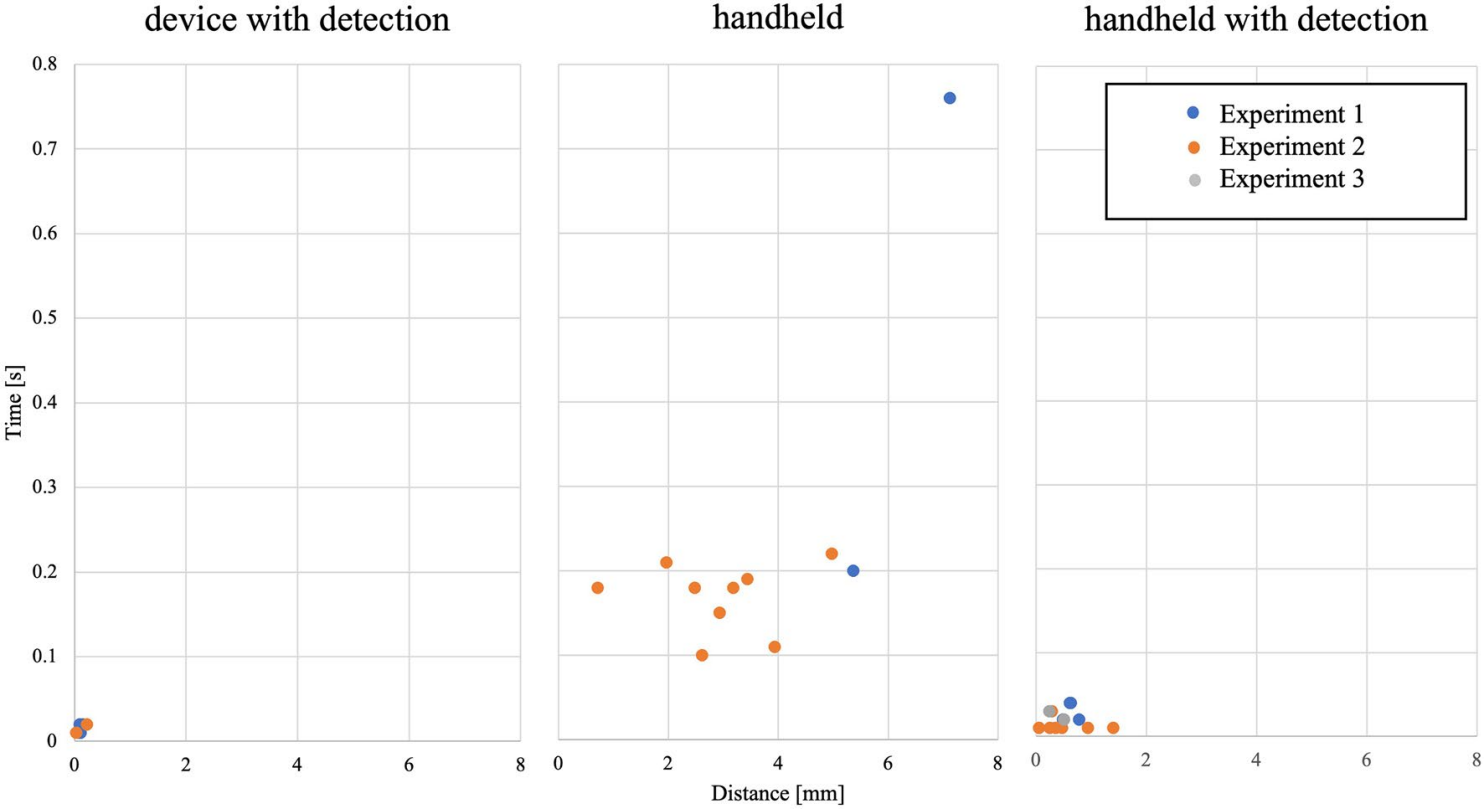
Time-distance plot of the haptic interface surgical drill with the penetration detection function and the handheld surgical drill with and without the penetration detection function. The average reaction time to detect penetration and distance after penetration were both significantly improved when a haptic interface surgical drill with the penetration detection function was used. The drill parameters used in each experiment are summarized in Table 1.

The average time to detect the penetration of lamina by the drill with the penetration detection function was 0.015 ± 0.005 s (range 0.01–0.02 s), and the travel distance after penetration was 0.11 ± 0.063 mm (range 0.03–0.22 mm, Table 1, Fig. 6). Notably, the extremely small range of the detection time and distance in each experiment demonstrates the high reproducibility of the custom-made drill. Additionally, the excellent correlation between the time to detect penetration and the travel distance after penetration indicate the accuracy of the device in this animal experience setting (r = 0.996, p < 0.001).

**Table 1:** The data described in this paper were obtained using three pigs. The drill parameters used in each experiment are listed in the table.

	Body weight (kg)	${\dot{F}}_{threshold}$ (N/s)	${\dot{x}}_{threshold}$(mm/s)	Drill speed (rpm)
Experiment 1	41	− 10	2	10,000
Experiment 2	36	− 7	2	30,000
Experiment 3	34	− 7	2	15,000

### The time to detect lamina penetration and the travel distance after penetration by the handheld surgical drill without the penetration detection function

Three spine surgeons drilled the posterior laminae 3 times each until they noted penetration by the custom-made haptic drill without the penetration detection function. The average reaction time was 0.226 ± 0.181 s (range 0.10–0.76 s), and the distance travelled after penetration was 3.52 ± 1.770 mm (1.96–7.12 mm, Fig. 6). No difference was observed among the participants for the average reaction time or the distance travelled after penetration (Fig. 6).

There was no correlation between the detection time and distance, indicating that bone drilling using a handheld drill was less reproducible (r = 0.045, p = 0.909). These results clearly showed that even in an experienced surgeon’s hand, it is difficult to recognize the penetration of the lamina early and to stop the drill immediately after detecting the penetration of the lamina during surgery.

There were statistically significant differences for both the average reaction time and the distance travelled after penetration between the handheld drill and the drill with the penetration detection function (p < 0.001). The penetration detection time of the device was approximately one-tenth as fast as that of the handheld drill, and the mean difference was 0.154 ± 0.024 s [95% CI 0.099, 0.209 s] and 2.862 ± 0.741 mm [95% CI 1.211, 4.514 mm].

### The time to detect lamina penetration and the travel distance after penetration of the handheld surgical drill with the penetration detection function

Similarly, three spine surgeons drilled the posterior laminae 3 times each until they detected the penetration by the custom-made haptic drill with the penetration detection function. The average reaction time was 0.020 ± 0.012 s (range 0.01–0.04 s), and the distance travelled after penetration was 0.541 ± 0.351 mm (range 0.06–1.40 mm, Fig. 6). The reaction time to detect penetration and the distance after penetration were both significantly improved when compared with those of the handheld surgical drill without the penetration detection function, with mean differences of 0.049 ± 0.019 s [95% CI 0.012, 0.086 s] and 2.511 ± 0.537 mm [95% CI 1.505, 3.516 mm], respectively.

### Penetration of laminae using a surgical drill with a haptic interface using horizontal positional information

Three spine surgeons drilled the posterior laminae 4 times each. Using the horizontal position information, it was set to automatically stop when the drill travelled 2 mm after penetration detection (Fig. 5B). The distance travelled after penetration detection was 2 mm in 10 cases, and overdetection occurred 2 times. The average distance after penetration using the horizontal position information was − 0.429 ± 0.202 mm (range − 1 to 0 mm). There was no significant difference between the distance travelled after penetration in the horizontal direction and the vertical direction, with a mean difference of 0.967 ± 0.269 mm [95% CI 0.382, 1.552 mm]. This confirmed the accuracy of penetration detection when the drill was operated in the horizontal direction.

## Discussion

In this study, we successfully conducted a performance evaluation test of a custom-made haptic interface surgical drill on miniature pigs. The reaction time until spine surgeons recognized penetration by the custom-made haptic drill without the penetration detection function was 0.10–0.22 s. In contrast, the time to detect the penetration of the lamina on the drill with the penetration detection function was 0.01–0.02 s with an extremely small error range of 0.005 s. We quantitatively demonstrated that a much faster automatic stop of a surgical drill can be achieved by the integration of haptic technology into spinal drills with a penetration detection function. Furthermore, the distance travelled after penetration with the penetration detection function was significantly shorter than that of the handheld drill, with excellent reproducibility. Therefore, we considered that the safety of the haptic drill was substantiated. We have also shown that it is possible to construct a simulator model with movement data, such as the reaction force, travel distance, travel speed, cutting torque and rotation speed of the drill. Therefore, we demonstrated that the differential signal of the abrupt changes in the reaction force and drilling speed is significant for improving the accuracy of the detection function and automatic stop function. We demonstrated the versatility of real haptic technology by evaluating a prototype of a haptic drill.

In this study, 3 spine surgeons drilled the posterior lamina of miniature pigs. Their years of experience differed, ranging from 2 to 22 years (2, 7, and 22 years). The average reaction time was 0.169 ± 0.041 s, and the distance travelled after penetration was 2.982 ± 1.242 mm when using a handheld drill without the penetration detection function. Additionally, the lack of correlation between the detection time and distance indicated that bone drilling using a handheld drill was less reproducible. However, when using the handheld surgical drill with the penetration detection function, the reaction time and the distance after penetration were both improved. The average reaction time to detect penetration and the distance after penetration were both significantly shorter than those of the handheld surgical drill without the penetration detection function, with mean differences of 0.049 ± 0.019 s and 2.511 ± 0.537 mm, respectively. This result clearly showed that even when the drill was used as a handheld device, the reaction time and the distance after penetration can be significantly shortened if it is equipped with real haptics.

Interestingly, analysis of the reaction time and the travel distance after penetration of the handheld surgical drill with the penetration detection function showed no statistically significant differences among the surgeons for their average reaction time and the distance travelled after penetration. According to this result, surgeons can recognize penetration extremely quickly with haptic drills regardless of their years of experience. As we have described in the introduction section, gaining surgical experience has thus far been considered very important in reducing the possibility of neurological complications caused by surgical drills. However, it is difficult to gain experience in actual spine surgery without years of experience performing spinal surgery. Therefore, the establishment of a surgical simulator using real haptics technology will be important for young spine surgeons to gain experience in order to avoid neurological complications caused by surgical drilling.

Several previous reports described the availability of haptic technology for surgical simulators^12–14^. Meyer et al. compared the drilling performance of a bone simulator with a haptic system between the trainee resident group and the expert staff surgeon group. They concluded that there were no significant differences in surgical time or the accuracy of the surgical technique^13^. Thus, the use of a spine surgery simulator with a haptic system may be useful for spine surgery education, which has a steep learning curve. Previous reports showed the utility of real haptics technology only for surgical simulators. This study is the first report indicating the accuracy of the penetration detection function and automatic stop function of haptic drills when performing real surgery.

In this study, we also proved the accuracy of penetration detection when the surgeon operated the drill in the horizontal direction, which is similar to the actual surgical procedure. It was possible to detect penetration according to the shape of the lamina by recognizing the position by dividing it into 2 mm increments. On the other hand, the position information of the lamina can be moved by the deviation of the spine during the procedure, so overdetection may occur; however, overdetection never causes damage to the spinal cord. Currently, we are planning to add alert systems to the penetration detection system. Further refinement of the penetration detection system will include minimization of the overdetection of penetration.

Furthermore, the concept of using real haptic technology and virtual reality (VR) for robotic surgery has also been developed^14,15^. These systems are controlled with a haptic device that feeds the exerted drilling force into the hand of the surgeon in real time. Thus, surgeons can detect the differences in tissues and then perform surgeries more safely and accurately. Real haptic systems are also considered to be an essential technology for the development of robotic telesurgery^16^.

We acknowledge the limitation that we were unable to use human cadavers due to resource limitations. In this study, the drill was used only in a vertical direction against the vertebral lamina of young miniature pigs. However, in actual surgery, the surgeon moves the drill in various directions against the vertebral lamina in various age populations while drilling. Further study for the improvement of this evolutional instrument may require the evaluation of the safety and utility of the haptic interface surgical drill in various bone conditions, including osteoporotic bone.

## Conclusion

In the present study, a prototype high-speed drill with a haptic interface successfully detected the penetration of the porcine posterior lamina more accurately and more reproducibly than experienced spine surgeons. The integration of haptic technology into spinal drills may be expected to have future applications in accurate, safe robotic surgery and telesurgery.

## Supplementary Information

Supplementary Video 1.

## Data Availability

The datasets used and/or analysed during the current study are available from the corresponding author upon reasonable request.

## References

[1] Imajo Y (2015). Japanese 2011 nationwide survey on complications from spine surgery. J. Orthop. Sci..

[2] Cammisa F (2000). Incidental durotomy in spine surgery. Spine.

[3] Kotilainen E, Valtonen S, Carlson C (1993). Microsurgical treatment of lumbar disc herniation: Follow-up of 237 patients. Acta Neurochir..

[4] Rampersaud Y (2006). Intraoperative adverse events and related postoperative complications in spine surgery: Implications for enhancing patient safety founded on evidence-based protocols. Spine.

[5] Stolke D, Sollmann W, Seifert V (1989). Intra- and postoperative complications in lumbar disc surgery. Spine.

[6] Wang J, Bohlman H, Riew K (1998). Dural tears secondary to operations on the lumbar spine. Management and results after a two-year-minimum follow-up of eighty-eight patients. J. Bone Jt. Surg. Am..

[7] Guerin P (2012). Incidental durotomy during spine surgery: Incidence, management and complications. A retrospective review. Injury.

[8] Ohnishi K, Saito Y, Fukushima S, Matsunaga T, Nozaki T (2017). Future society opened by real haptics. J. Jpn. Soc. Appl. Electromagn. Mech..

[9] Ohnishi K (2017). Real haptics and its applications. IEEJ.

[10] Takuya, M. *et al.* Multi functional drill incorporating linear motor for haptic surgical instrument and simulator. In *2021 IEEE International Conference on Mechatronics (ICM)* (2021).

[11] Kobayashi, H. *et al.* Development of orthopedic haptic drill for detection of penetration. In *The 7th IEEJ International Workshop on Sensing, Actuation, Motion Control, and Optimization* (2021)*.*

[12] Ha-Van Q, Schwendinger H, Kim Y, Harders M (2020). Design and characterization of an actuated drill mockup for orthopedic surgical training. IEEE Trans. Haptics..

[13] Meyer C, Noda F, Folsom C (2020). Hybrid surgical simulator: A temporal bone simulator validation study of the Stryker surgical simulator (S3). Mil. Med..

[14] Chen X, Sun P, Liao D (2018). A patient-specific haptic drilling simulator based on virtual reality for dental implant surgery. Int. J. Comput. Assist. Radiol. Surg..

[15] Robotic spinal surgery system with force feedback for teleoperated drilling—Rezazadeh—2019. *J. Eng. Wiley Online Libr*. 10.1049/joe.2018.9407 (2021).

[16] Matthew SG, Joseph DL, Srikanth ND, Dhruv KCG, Gregory DS (2019). Robot. Spinal Surg..

